# Global Perspective on Acute Kidney Injury in Nepal

**DOI:** 10.34067/KID.0000001018

**Published:** 2025-10-02

**Authors:** Deepak Sharma, Taranath Sharma, Jie Tang

**Affiliations:** 1 Department of Internal Medicine and Nephrology, Dhulikhel Hospital, Kathmandu University Hospital, Dhulikhel, Nepal; 2 Department of Internal Medicine, College of Medical sciences, Kathmandu University, Bharatpur, Nepal; 3 Division of Kidney Diseases and Hypertension, Alpert Medical School of Brown University, Providence, Rhode Island

**Keywords:** AKI, drug nephrotoxicity, epidemiology and outcomes, health equity, diversity, and inclusion, leptospirosis, risk factors, KRT

## Introduction

Nepal has a population of over 30 million, with approximately 33.83% residing in rural areas.^1^ Mountainous areas particularly have limited access to tertiary care centers, which are concentrated primarily in urban hubs such as Kathmandu and Pokhara. In the context of AKI, delays in receiving care are common due to the lack of early detection and transportation. Patients in remote districts may have to travel 6–24 hours just to access basic laboratory tests or dialysis services.

A dual health care system comprising both public and private sectors offers subsidized or free services. However; it faces challenges such as underfunding, staff shortage, and overcrowding. Private facilities often deliver higher quality care but are concentrated in urban areas and require out-of-pocket payments, making them inaccessible to many, and limiting acute care delivery, especially in rural areas. AKI is an underrecognized and under treated disease entity that is strongly associated with increased morbidity and mortality, particularly in critically ill and rural populations.^2^ This article provides an overview of AKI in Nepal, highlighting gaps in epidemiology, health system response, and opportunities for improvement.

## Epidemiology of AKI in Nepal

The lack of national surveillance and reliance on single-center studies make it difficult to accurately quantify AKI incidence. Regional studies have reported an AKI incidence of 15% in non–intensive care unit admissions and up to 60% among intensive care unit admitted patients.^2,3^ There appears to be substantial regional heterogeneity in AKI burden, likely reflecting differences in health care access, disease burden, and diagnostic capabilities. Studies in low- and middle-income countries (LMICs) indicate that community-acquired AKI is more common, likely due to the lack of public awareness, delayed presentation, comorbidity burden, and limited access to care.^4^ Factors linked to AKI include infections, hypovolemia from dehydration, and obstetric complications. Nepal faces a “double burden” of disease, with a rising prevalence of noncommunicable conditions such as hypertension and diabetes,^5^ alongside ongoing threats from infectious diseases. This epidemiologic transition heightens both the risk and complexity of AKI in Nepal, particularly in vulnerable populations as in most of LMICs.

## Etiology and Risk Factors

Most cases of AKI in Nepal are community-acquired AKI and are strongly linked to preventable and infectious etiologies. Sepsis is a major contributing factor, with tropical infections frequently implicated. Diarrheal illnesses often lead to severe dehydration and hypovolemic shock, particularly in children. Acute poststreptococcal GN remains a significant cause of AKI in both children and adults. Nephrotic syndrome and severe malaria tend to occur together in the Terai region, and both conditions can independently contribute to the development of AKI. Snakebite envenomation is another common cause of AKI in the southern lowlands and Terai region bordering India. It often results in prolonged hospitalization, severe AKI requiring dialysis, and high mortality if treatment is delayed.^6^ Ingestion of nephrotoxic herbal remedies also contributes to the AKI disease burden, as Ayurveda is practiced heavily. Common traditional remedies in Nepal include preparations containing nephrotoxic aristolochic acid, although formal pharmacovigilance is lacking.^7^ Obstetric complications such as preeclampsia, eclampsia, and sepsis related to unsafe deliveries or abortions remain major causes of AKI.^8^ Finally, hospital-acquired AKI, although less common, occurs due to sepsis and drug toxicities.^9^

## Diagnosis and Management

In Nepal, AKI diagnosis often relies on symptom-based risk assessment and point-of-care creatinine and urine dipstick testing due to limited lab facilities. Ultrasonography is used when available to assess chronicity and exclude postrenal causes. The International Society of Nephrology 0by25 trial, which included Nepalese patients, found that community-level point-of-care testing improved early detection, guided fluid management, and reduced unnecessary hospitalizations, although challenges remain with limited resources and inadequate follow-up.^4^

AKI management in Nepal is largely supportive, focusing on early detection, treatment of underlying causes, volume resuscitation, and medication dose adjustment. Protocol-based care and provider training have improved patient outcomes and reduced unnecessary admissions in urban areas.^9^ KRT, mainly intermittent hemodialysis, is generally limited to tertiary care centers and is reserved for patients meeting guideline-based indications such as refractory hyperkalemia. Continuous KRT is available only in select facilities, underscoring the need for early diagnosis, protocol-driven care, and context-specific strategies aligned with guidelines for LMICs.^10^

## Health System Challenges

1. Limited Access to KRT: The number of public facilities offering either hemodialysis or peritoneal dialysis is insufficient, and the high out-of-pocket costs at private centers make dialysis treatment unaffordable for most patients. Kidney transplantation is also limited, accessible to only a small number of patients, with services largely concentrated in the capital.^10^
2. Workforce Shortages: Currently, there are fewer than ten nephrologists per million people, along with a shortage of dialysis nurses and other supporting staff.
3. Diagnostic and Surveillance Gaps: Access to essential diagnostics such as laboratory testing, kidney biopsy, and imaging is limited, especially at the primary care level. Reliable surveillance systems are also desperately needed.
4. Financial Barriers: The lack of health insurance or public reimbursement for both KRT and essential medications leads to high out-of-pocket expenditures.
5. Inadequate Disease Screening, Prevention, and Follow-up: Kidney disease screening is not performed in the community setting, despite the rising prevalence of diabetes and hypertension. Even after diagnosis, the lack of structured follow-ups leads many patients with AKI to suffer irreversible organ damage.
6. Geographical and Socioeconomic Disparities: Rural areas face greater barriers to health care due to challenging terrain, poverty, and gender inequities in access to care.
7. Research and Policy Limitations: In Nepal, there has been a limited understanding of population-specific epidemiology and clinical characteristics due to inadequate research capacity. Consequently, a well-defined disease management strategy has been lacking.

## Economic Impact

AKI imposes substantial economic burdens through prolonged hospital stays, intensive care, and dialysis. It also predominantly affects working-age adults, leading to losses in income and productivity for society.

## Recommendations

Improving AKI management in Nepal depends on targeted interventions at the health system, provider, and community levels. Key recommendations include:

1. Implement point-of-care diagnostics for timely AKI diagnosis and management.
2. Implement standardized AKI protocols and promptly address underlying causes, tailored to local epidemiology and available resources.
3. Advocate for affordable diagnostics, access to essential medications, KRT supplies, and develop national guidelines and surveillance systems to monitor AKI.
4. Expand peritoneal dialysis, a cost-effective KRT modality for AKI; programs such as Saving Young Lives demonstrate both the feasibility and effect of interventions.
5. Expand health care workforce and optimize training through international collaborations and mentorship programs.
6. Conduct public campaigns using materials adapted to local literacy to raise disease awareness and improve early AKI detection.
7. Set up effective community-based interventions to manage key risk factors of AKI and encourage timely referral to a kidney specialist.
8. Promote epidemiologic research on AKI incidence, outcomes, and care barriers, focusing on context-specific interventions, long-term outcomes, and cost-effectiveness to guide policy.

## Conclusions

AKI is a major health care challenge in Nepal, driven by causes including infections and obstetric complications. Although progress has been made, gaps remain in awareness, surveillance, infrastructure, and policy. Addressing these challenges requires strengthening the health care workforce, expanding access to affordable diagnostics and treatment, promoting community awareness and disease prevention, and integrating AKI into national health programs to achieve equitable kidney health.
